# Desmoid-type fibromatosis of the breast: a case report and literature review

**DOI:** 10.3389/fonc.2025.1482024

**Published:** 2025-02-11

**Authors:** Zijun Zhao, Qingyao Shang, Chenxuan Yang, Jiaxiang Liu, Shanqing Liu, Xiaoqian Li, Xiyu Kang, Jiaxian Yue, Xin Wang, Xiang Wang

**Affiliations:** ^1^ Department of Breast Surgery, National Cancer Center/National Clinical Research Center for Cancer/Cancer Hospital, Chinese Academy of Medical Sciences and Peking Union Medical College, Beijing, China; ^2^ Department of Breast Disease, The Affiliated Cancer Hospital of Zhengzhou University and Henan Cancer Hospital, Zhengzhou, China; ^3^ Department of General Surgery, Beijing Huairou Teaching Hospital, Capital Medical University, Beijing, China

**Keywords:** desmoid-type fibromatosis, breast, case report, pathology, treatment

## Abstract

Breast desmoid-type fibromatosis (BDF) is a rare tumor predominated by mesenchymal cells. It has a high recurrence rate, although distal metastasis is uncommon. It resembles breast cancer clinically, and histological pathology is the only approach to a confirmed diagnosis. Comprehensive and individualized treatments were recommended for BDF patients. Here, we presented a case of BDF secondary to primary breast carcinoma in our center. A 47-year-old female complained of a large mass in her left breast for 2.5 months. She has a past history of left breast carcinoma with a failure of surgical and systemic intervention. Despite an active re-operation, she still suffered from disease progression with a bad prognosis. After our report, the clinicopathological traits, differential diagnosis of BDF and current recommendation of management were discussed. This case report aimed to make a clear recognition of this rare and aggressive disease and elaborate up-to-date treatment recommendations. More effective drugs and larger sample clinical studies are encouraged for better management of refractory and progressive BDF.

## Introduction

Desmoid-type fibromatosis (DF) is a rare type of mesenchymal tumor that originated from musculoaponeurotic tissues with an annual incidence rate of four to five cases per million individuals (1, 2), accounting for 0.03%–0.1% of solid tumors and 3% of mesenchymal tumors (3). Although the tumor rarely causes distal metastasis, local progress is aggressive and the overall recurrence rate is high after surgery with the total recurrence rate ranging from 18% to 39% (4–8). For a complete excision, the local recurrence rate could be controlled approximately 7%–28% (4, 5, 7, 9–13), while an incomplete excision could lead to a higher rate (26%–100%) (4, 5, 7, 9, 11). The most vulnerable group of patients is middle-aged women (mean age ranging from 37 to 50.3 years) (14–20). DF consists of three forms: intra-abdominal DF, abdominal wall DF, and extra-abdominal DF. Intra-abdominal subtype presents sporadically, and certain types of them are related to familial adenomatous polyposis (FAP) or Gardner syndrome (21, 22). The extra-abdominal subtype is formed by a rare type of monoclonal fibroblastic proliferation with local aggressiveness and a remarkable tendency to recur (23). It usually occurs in the head and neck, chest wall/paraspinal region, shoulder, hip/buttock region, and extremities (24, 25). Breast desmoid-type fibromatosis (BDF) is a subtype of extra-abdominal form of DF, accounting for about 4% in extra-abdominal DF and less than 2‰ in all breast tumors (17, 26–28). Bilateral and multicentric BDF are rarer, accounting for merely 4% of BDF cases (29, 30). According to previous literature, history of trauma and operation are clinical risk factors of DF, accounting for about one-third of all DF-related causes (25, 26, 31). As the incidence of DF in female patients is twice of that in the male counterpart (3), some risk factors specifically related to women are essential in the development of DF. For instance, breast implants are a potential risk factor in BDF (32) and estrogen also has a potential impact on the pathogenesis of DF, especially in pregnant women (27, 33). Genetically, mutation of catenin beta 1 (CTNNB1) and mutation of Adenomatous Polyposis Coli gene (APC) occurred in 80%–85% and 5%–15% of DF cases, respectively. The latter mutation usually occurred in intra-abdominal DF as a familial disorder related to FAP (3).

Because of similarities between BDF and breast carcinoma in clinical examination and features of medical imaging, it is difficult for clinicians to differentiate it from breast carcinoma (28). Pathology is the gold standard for confirming diagnosis of DF. Of notice, a pathological professional experienced in diagnosing mesenchymal tumors is necessary for proofreading of the pathological results (17, 27, 34).

Here, we presented a case of aggressive and refractory BDF in a middle-aged female with a gloomy prognosis. Unlike the typical BDF, the patient quickly suffered from “locoregional and distal relapse” with severe symptoms even though a complete mastectomy was performed. After the case report, we discussed the clinical and pathological features, differential diagnosis, and up-to-date treatment recommendations of BDF. Because of its rarity, few medical practitioners have a clear perception of BDF. Our goal is to present a detailed elaboration of BDF and let medical practitioners be aware of the danger of this disease. Pathological diagnosis is the key to this disease, and timely and appropriate management for BDF is crucial for the patient’s response, even for the prognosis. More effective drugs are urgently needed, especially for refractory and aggressive cases.

## Case report

**Figure 1:**
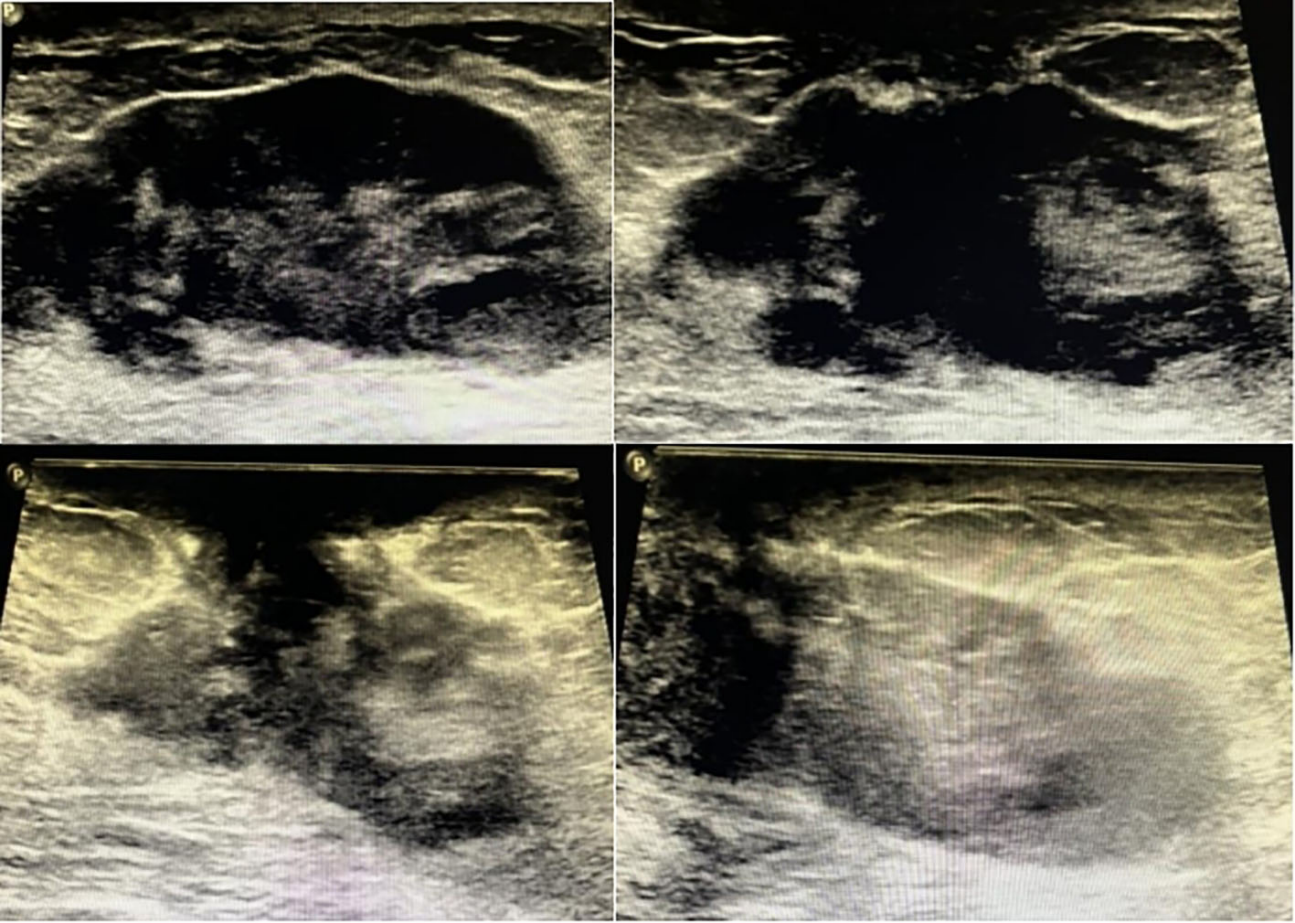
Sonography of the patient of breast desmoid-type fibromatosis in different sections of scan.

**Figure 2:**
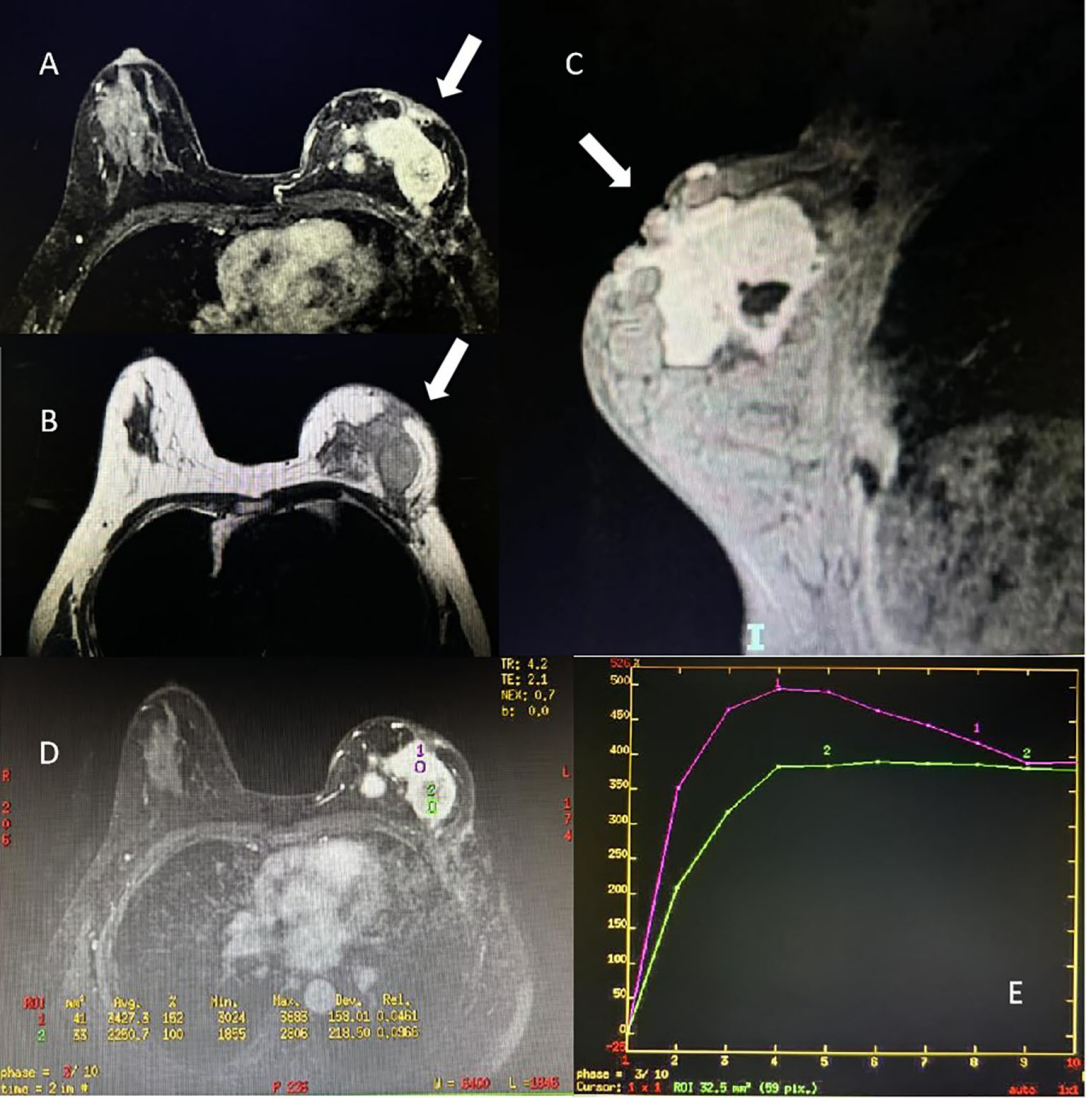
Magnetic resonance imaging of the patient of breast desmoid-type fibromatosis (white arrow). **(A)** Axial view of contrast enhancement of T1 signal. **(B)** Axial view of T2 signal. **(C)** Sagittal view of the mass of right breast. **(D, E)** Time-signal intensity curve of the pattern of contrast enhancement in region-of-interest.

A 47-year-old Chinese female was admitted to our hospital because of a large lump in the left breast for 2.5 months. Eight months ago, she was diagnosed with “breast cancer” of the left breast in a local hospital and underwent breast-conserving surgery. Paraffin pathology suggested “metaplastic carcinoma, 3 cm × 2 cm × 2 cm, no metastatic lymph nodes, Ki67 50%, Vimentin (+), S-100 (-), Desmin (-), ER (-), PR (-), HER2 (-).” The pathological staging is IIA, T2N0(sn). Subsequently, the patient received six cycles of TEC (docetaxel + epirubucin + cyclophosphamide) adjuvant chemotherapy. Shortly after the chemotherapy, the patients found a relapse of tumor in the left breast without apparent pain, nipple discharge, nipple inversion, skin depression, or even redness. The patient transferred to the outpatients in our hospital for treatment. The ultrasound found multiple hypoechoic large lumps fused over the region from 11 o’clock to 6 o’clock, with the largest one measuring 6.6 cm × 2.2 cm. The echo was granular and nodular without any significant vascularity (**Figure 1**). Magnetic resonance imaging (MRI) suggested multiple fusions of skeptical malignancies with skin involvement in the left breast with an impression of birads V. The largest one of which was 5.7 cm × 3.2 cm with unclear margins. T1WI signal was hypointense, and T2WI signal was hyperintense. The breast lesion demonstrated heterogeneously rapid contrast enhancement that plateaued on delayed phases consistent with a type-three curve (**Figure 2**). No other abnormal findings were found in blood tests (multiple tumor biomarkers), CT scans of the chest, or abdominal and breast/axillary lymph node ultrasounds. Then an ultrasound-guided fine needle biopsy was performed, and the result suggested “skeptically fibro-epithelial neoplasm.” For further treatment, the patient was referred to our department and received an excisional biopsy of this lesion in the left breast, and the paraffin pathology indicated it as “desmoid-type fibromatosis.” After diagnosis, the patient was appointed to our clinic and a further plan of the treatment was discussed. After careful consideration and discussion, the patient and her relatives decided to receive left breast mastectomy. The final result of paraffin pathology indicated the same as the previous one. It was a pity that the patient did not return to our hospital for a thorough review and a complete follow-up as requested by the appointment. After 4 months, the patient suffered from “chronic pain in her left chest wall and then in her whole body.” Radiological examinations in the local hospital suggested “local and distal relapse of tumor (the tumor relapsed in the left chest wall with lung metastasis and a massive amount of ascites).” (Details were not available; the information was told by her relative via telephone contact). Unfortunately, the patient only received palliative and supportive treatment in the local hospital. She died five months after the second operation.

## Discussion

In clinical examination, BDF usually presents as a palpable, solitary, painless, firm, and mobile mass, adhering or not to the chest wall or sometimes accompanied with skin and nipple retraction (19, 20, 35, 36). In mammography, the tumor can be identified only in one-third of the cases (28). It was demonstrated as an irregular, lobular, noncalcified, and spiculated mass with spiculated margin and a high density, which is similar to breast carcinoma (29, 37). In the breast ultrasound, it was usually a solid, fusiform, spiculated or microlobulated, and hypoechoic masses in an irregular shape with posterior attenuation. The lesion tends to grow along muscle fibers and fascia. Necrosis and calcification is rare in BDF (24). Sometimes the benign image can also be presented (28, 37). For cases of locally aggression and infiltration of the BDF, deformation and/or damage of the Cooper’s ligaments, involvement of the skin, nipple-areola complex, and pectoralis muscle could be demonstrated (29, 30). MRI of the breast showed an ill-defined, spiculated, and lobulated mass in which T1-weighted image was hypo-to-iso-intensed compared with the image of muscle while T2-weighted image was hyperintensed (38–41). Patterns of dynamic enhancement of the mass vary and mainly demonstrate as a gradual enhancement (37, 39), revealing a considerable proportion of non-enhancement of collagenous tissue and myxoid change of the lesion (42). For some cases mimicking that of an invasive carcinoma of breast, a type-three curve (rapid enhancement and washout on dynamic MRI) can be displayed, which is tough for medical workers to differentiate from typical breast cancer (43).

Pathologically, BDF is an locally aggressive monoclonal proliferation of infiltrative fibroblasts and myofibroblasts (44). The tissue of BDF is rich in spindle cells, forming many of long and uniform fascicles and extending into the surrounding fibrous stroma. Especially, some fat tissues are entrapped and lymphocytes are presented at the periphery of the lesion (34). The normal tissue can be encompassed by the lesion (45). No nuclear atypia is in the fibromatosis cells and the cell nuclei are characteristically bland and spaced (46–50). Regarding to immunohistochemistry (IHC), positive results of Vimentin, β-catenin nuclear staining, and smooth muscle actin (SMA) cytoplasmic staining can be detected in fibromatosis cells whereas staining of S-100, CD34, CKs, and p63 are usually negative (34, 51).

Because it is tough to differentiate between BDF and other breast tumors based on clinical features, pathological diagnosis is the predominant approach. Histologically, BDF should be differentiated from fibromatosis-like metaplastic breast carcinoma (MBC), scar tissue, and other breast spindle cell lesion (BSCL). For fibromatosis-like MBC, its histologic features include focal cytological atypia and areas of epithelioid differentiation on morphology (34). Usually, infiltration of inflammatory cells and with lymphoid follicles could appear in tissue of fibromatosis-like MBC. Moreover, nuclear spacing was characteristically rare in the lesion. The IHC result usually indicated positive results of CK, p63, and SMA and negative results of CD34, hormone receptor and HER-2. Scar is a common type of BSCL. Identification of a scar lesion could be helped by a history of trauma, operation and some special pathological traits including existence of fat necrosis, foamy macrophages, foreign body giant cells, and hemosiderin deposition (34). Some fresh scars are featured by reactive spindle cell nodules while mature scars are more similar to fibromatosis or fibromatosis-like MBC (34). Speaking of IHC, the result of staining of CKs, desmin, p63, CD34, and nuclear β-catenin on IHC are all negative while CK expression is rich in lesion of fibromatosis-like MBC and BDF. Scar is rich in SMA expression. Other BSCLs also include myofibroblastoma and nodular fasciitis (NF). Regarding myofibroblastoma of the breast, it is a tumor featured by myofibroblastic but without epithelial component (52). Incidence of myofibroblastoma in male is higher than that in female population. Myofibroblastoma is enriched in spindle cells in fascicular pattern intervened by of collagen fibers. On the contrary, it lacks of breast ducts and lobules (34). For IHC, positive results are presented in the staining of Bcl2, CD10/34/99, desmin, ER, PR, SMA while negative results in the staining of CKs, p63, and Rb (53). Different from the above types of lesion, NF is a self-limiting mass usually accompanied by symptom of pain or tender because of its fast growing. In the lesion of NF, a rapid clonal proliferation of bland-appearing stellate fibroblasts are arranged in a loose fascicular to a storiform pattern. NF has a variable cellularity with diversity of extracellular matrix (collagenous, myxoid, etc.). Another characteristic of NF is extravacation of RBCs. For IHC, NF is positively stained with actin whereas negatively stained in CK and p63 (53). The summary of the above differential diagnosis is shown in **Table 1**.

**Table 1:** Differential diagnosis of breast desmoid-type fibromatosis.

Tumor type	BDF	Fibromatosis-like MBC	Scar	Myofibroblastoma	NF
Histology	Infiltrative fibroblasts and myofibroblasts	Focal cytological atypia and areas of epithelioid differentiation	Fat necrosis and foamy macrophage	Rich in spindle cells in fascicular pattern intervened by collagen fibers	Rapid clonal proliferation of bland-appearing stellate fibroblasts and extravacation of red blood cells
Lymphocyte	+	+	–	NA	NA
Nuclear atypia	–	+	–	–	–
Cell nuclei	Bland and spaced	Nuclear spacing is rare	NA	No regularly spaced nuclei	Plump vesicular nuclei
IHC					
SMA	+	–	+	+	NA
CD34	–	–	–	+	NA
CK	–	+	–	–	–
p63	–	+	–	–	–
β-catenin	+	NA	–	NA	NA

BDF, breast desmoid-type fibromatosis; CK, cytokeratin; IHC, immunohistochemistry; MBC, metaplastic breast carcinoma; NA, not available; NF, Nodular fasciitis; SMA, smooth muscle actin.

According to 2024 National Comprehensive Cancer Network (NCCN) clinical practice guidelines in soft tissue sarcoma (version 1) (54), treatments for DF include: surgical operation (including ablation/embolization), radiotherapy, and systemic therapy. Choice and sequence of the treatment may depend on medical history (prior treatment, recurrence or not), location and size of tumor, potential morbidity of the therapeutic option, and patient preference. In general, surgery is not necessarily the first-line treatment option. It is considered as a primary treatment if the patient is operable by a multidisciplinary tumor board. According to UpToDate website of DF, surgery is necessary in following situations: Symptomatic lesion; enlargement of lesion; threatening to surrounding essential anatomic structures (large vessels, important organs, etc.) (51). If the surgery achieves R0 resection (negative margin) or radiographic response achieved, only observation may be necessary and adjuvant radiotherapy can be planned. If the surgery resulted in R1 resection (positive margins microscopically or minimal residual disease), careful observation may be a choice for some low-risk patients (asymptomatic, stable, location is not associated with potential functional limitation). If necessary, re-resection, adjuvant radiotherapy, and systemic therapy can be choices. For R2 resection (macroscopic margins), or condition where surgery will cause significant morbidity and patients’ refusal of surgery, then the choice of subsequent treatment include: definitive radiotherapy, systemic therapy, radical surgery, ablation or observation (51). The radiotherapy is primarily recommended for non-mesenteric desmoid tumor but not in retroperitoneal/intra-abdominal desmoid tumors. It is also indicated where recurrent lesion is tough to be completely resected (R1/R2 resection), or the patient fails to tolerate or respond well to systemic therapy. The recommended dose and fraction of definitive radiotherapy is 56 Gy in 28 fractions (55). In some circumstances, definitive or adjuvant radiotherapy with a dose larger than 50–56 Gy may be beneficial, administered in standard 1.8–2 Gy/fraction post-operatively (24-26) (56–58). Speaking of systemic therapy, the preferred regimens for advanced and inoperable DF consist of non-cytotoxic agents for slightly-to-moderately serious symptoms (Nirogacestat, Sorafenib, Pazopanib, Imatinib) and cytotoxic agents for serious symptoms (Methotrexate and vinorelbine, Methotrexate and vinblastine, Liposomal doxorubicin, and Doxorubicin ± dacarbazine) (59–69). Among non-cytotoxic agents, agents of targeted therapy take the largest part. Presently, targeted therapy of DF include tyrosine kinase inhibitors (TKIs), Wnt/β-catenin inhibitors, and γ-secretase inhibitors (GSI) (70). TKIs consist of imatinib, sorafenib, sunitinib, pazopani, and anlotinib. In clinical practice, imatinib can inhibit the tumor progression and it is commonly used after failure of previous lines of DF treatment. According to previous studies, sorafenib could significantly improve the prognosis of patients with refractory and progressive DF (60). Other studies also found potential treatment effects of sunitinib, pazopani, and anlotinib (70–72). Tegavivint, also known as BC2059, is a representative of Wnt/β-catenin inhibitors. It serves as a selective inhibitor of nuclear β-catenin through binding TBL-1. It is right now the only drug that is under evaluated in a phase I clinical trial (NCT03459469) recruiting DF patients (70, 73). One preclinical study has found a potential capability of Tegavivint in nuclear β-catenin inhibition, which is an essential anti-tumor effect of the drugs in treating DF patients with CTNNB1 mutation (73). For certain situations, Sulindac or other nonsteroidal anti-inflammatory drugs (NSAIDs), including celecoxib can be used (74). NSAIDs are non-selective inhibitors of cyclooxygenase-2 (COX-2), involved in Wnt/β-catenin pathway (36). CTNNB1 gene encodes β-catenin and CTNNB1 mutation is common in extra-abdominal DF (36, 75). In normal cells, wide-type β-catenin can be phosphorylated by two kinases (GSK3 and CK1γ) and then ubiquitylated and destroyed by proteasome (76). As mutation of β-catenin occurs, phosphorylation of β-catenin is prohibited which triggers β-catenin accumulation and subsequent translocation of β-catenin towards nucleus to activate the transcription of target genes including COX-2 (77). COX-2 is a typical pro-tumor molecule that gets involved not only in promoting cell proliferation, migration, and angiogenesis but also in prohibition of apoptosis (77). Hence, NSAIDs can suppress the function of this downstream molecule to inhibit the growth of tumor. GSI is a brand-new type of targeted therapy for DF. PF-03084014 is the representative of GSI and is under investigation for its efficacy for DF patients. The characteristic of this drug is that it can merely inhibit tumor progression but it is not a killer of tumor cell. It basically gets involved in Notch pathway, where it can directly or indirectly interacts with Wnt/APC/β-catenin pathway, affecting growth and development of DF cells (70). Some early phase clinical studies had demonstrated potential effect of PF-03084014 in refractory and progressive DF (78, 79). Hormonal or biological agents such as tamoxifen, toremifene, or low-dose interferon are also recommended choices. Cardiovascular events should be considered when Celecoxib is used (54). During the follow-up, patients should be regularly evaluated with CT or MRI for every three to 6 months in the first two to three years. Ultrasound examination can be considered for some location of the tumor. According to Uptodate website about DF (51), re-examination is suggested for every 6 months in the first 3 years, every 12 months in the second three years, and then one time in every 2 years.

For our report, this is a rare and aggressive case in our hospital. The most remarkable feature of this case was that the BDF lesion was refractory to either systemic therapy or surgical treatment (no matter tumor excision or mastectomy). Especially after the second operation, the tumor progressed rapidly and the patient complained severe pain and suffered from a wide spread of the tumor. Radiological examinations suggested local and distal metastasis (tumor in the left chest wall, lung metastasis, and ascites), which is surprising because a typical DF does not usually have a tendency of distal relapse. Nevertheless, the actual cause of the death of this patient was not available because the patient did not return to our hospital to complete a close follow-up. The only information of the condition of this patient was related by her relative through telephone. No more objective detail can be provided to support the information above. Moreover, whether or not the tumor in the latest relapse is still purely BDF is unknown because pathological diagnosis can no longer be acquired from this patient. But what is certain is that a non-specific, supportive, and palliative regimen for this patient is not helpful to ameliorate her symptom and prognosis. Unfortunately, shortage of large-scale clinical study to examine the efficacy of the new drugs limited the application of these novel techniques to save the lives of patients with refractory and progressive BDF. As a result, this problem should be urgently solved.

## Conclusion

BDF is a rare condition of breast tumor, but in some patients, it is very aggressive and the prognosis is poor. Although a low likelihood of distal metastasis, it has a high recurrence rate and sometimes responses poorly to conventional regimens for typical breast malignancies. It is tough for medical professionals to cope with this disease because a successful management of BDF not only needs an accurate diagnosis of pathology but also needs an appropriate and timely therapy for this disease. For refractory, progressive, and inoperable BDF, systemic therapy is indispensable. Scientific explorations of gene mutations and molecular interactions in BDF and large-sample clinical trials are important for development of new drugs with more powerful efficacy and less side effects.

## Data Availability

The raw data supporting the conclusions of this article will be made available by the authors, without undue reservation.
